# Standardized Description of the Feature Extraction Process to Transform Raw Data Into Meaningful Information for Enhancing Data Reuse: Consensus Study

**DOI:** 10.2196/38936

**Published:** 2022-10-17

**Authors:** Antoine Lamer, Mathilde Fruchart, Nicolas Paris, Benjamin Popoff, Anaïs Payen, Thibaut Balcaen, William Gacquer, Guillaume Bouzillé, Marc Cuggia, Matthieu Doutreligne, Emmanuel Chazard

**Affiliations:** 1 Univ. Lille CHU Lille ULR 2694 - METRICS: Évaluation des Technologies de santé et des Pratiques médicales Lille France; 2 Fédération régionale de recherche en psychiatrie et santé mentale (F2RSM Psy), Hauts-de-France Saint-André-Lez-Lille France; 3 InterHop Rennes France; 4 Department of Anaesthesiology and Critical Care Rouen University Hospital Rouen France; 5 Medical Information Department Amiens-Picardy University Hospital Amiens France; 6 Digital Services Department Amiens-Picardy University Hospital Amiens France; 7 Institut national de la santé et de la recherche médicale (INSERM), LTSI-UMR 1099 Univ Rennes, CHU Rennes Rennes France; 8 Mission Data Haute Autorité de Santé Saint-Denis France; 9 SoDa project team National Institute for Research in Digital Science and Technology (INRIA), Saclay-Île de France Gif-sur-Yvette France

**Keywords:** feature extraction, data reuse, data warehouse, database, algorithm, Observation Medical Outcomes Partnership

## Abstract

**Background:**

Despite the many opportunities data reuse offers, its implementation presents many difficulties, and raw data cannot be reused directly. Information is not always directly available in the source database and needs to be computed afterwards with raw data for defining an algorithm.

**Objective:**

The main purpose of this article is to present a standardized description of the steps and transformations required during the feature extraction process when conducting retrospective observational studies. A secondary objective is to identify how the features could be stored in the schema of a data warehouse.

**Methods:**

This study involved the following 3 main steps: (1) the collection of relevant study cases related to feature extraction and based on the automatic and secondary use of data; (2) the standardized description of raw data, steps, and transformations, which were common to the study cases; and (3) the identification of an appropriate table to store the features in the Observation Medical Outcomes Partnership (OMOP) common data model (CDM).

**Results:**

We interviewed 10 researchers from 3 French university hospitals and a national institution, who were involved in 8 retrospective and observational studies. Based on these studies, 2 states (track and feature) and 2 transformations (track definition and track aggregation) emerged. “Track” is a time-dependent signal or period of interest, defined by a statistical unit, a value, and 2 milestones (a start event and an end event). “Feature” is time-independent high-level information with dimensionality identical to the statistical unit of the study, defined by a label and a value. The time dimension has become implicit in the value or name of the variable. We propose the 2 tables “TRACK” and “FEATURE” to store variables obtained in feature extraction and extend the OMOP CDM.

**Conclusions:**

We propose a standardized description of the feature extraction process. The process combined the 2 steps of track definition and track aggregation. By dividing the feature extraction into these 2 steps, difficulty was managed during track definition. The standardization of tracks requires great expertise with regard to the data, but allows the application of an infinite number of complex transformations. On the contrary, track aggregation is a very simple operation with a finite number of possibilities. A complete description of these steps could enhance the reproducibility of retrospective studies.

## Introduction

The increasing implementation of electronic health records over the last few decades has made a significant amount of clinical data available in electronic format [1,2]. Originally, electronic health records were designed to collect and deliver data for health care, administrative, or billing purposes. In addition to these initial uses, they also offer opportunities for data reuse defined as “nondirect care use of personal health information” [3]. Thus, data reuse provides possibilities for research, quality of care assessment, activity management, or public health management [4-10].

When conducting research, the traditional approach consists of prospectively and often manually collecting simple and specific data according to the question addressed by the research protocol, using a clinical report form [11]. These data correspond to inclusion criteria and variables, that is, outcomes (eg, the length of stay in hospital or survival), exposures (eg, the taking of a drug or a surgery procedure), and adjusting variables (eg, age, sex, and patient history). When performing a prospective study, these data are defined upstream and are then collected manually in routine practice with human expertise, one record at a time, and background is taken into account. If needed, third-party data sources can be queried or caregiver expertise can be sought. This approach is expensive and time-consuming, and it generally results in only a limited sample size for a single use [7,11]. However, the final data set consists of explicit information that does not need further computation.

In contrast, data reuse builds on data sources already available at a low cost and offers a large volume of data [7]. Despite the many opportunities data reuse offers, its implementation presents many difficulties, and primary data cannot be reused directly. First, data reuse encounters data quality problems that arise from the manner in which the data were entered or collected [12-16], and it requires a phase of data cleaning to deduplicate, filter, homogenize, or convert raw data [17,18]. Moreover, information is not always directly available in the source database and needs to be computed later from raw data when defining an algorithm [19-23]. This is generally called “data transformation” [24], “data aggregation” [25,26], or “feature extraction” [27]. Even if feature extraction often approximately answers the question, the process is not easy and brings methodological issues. Indeed, features are extracted from a static database (already saved and closed) for patients for whom the care event has already been completed years earlier and for a large number of records. All scenarios must be taken into consideration so as to avoid having to modify the extracted records individually and by hand before the analysis. The method of extraction may have substantial effects on the features generated [28].

Lastly, the heterogeneity of local data models and vocabularies complicates the pooling of data and the sharing of algorithms, tools, and results [29-33]. Initiatives have emerged to promote the reuse of data through “large-scale clinical data sharing and federation” and the implementation of common data models (CDMs) [34-38]. Observational Health Data Sciences and Informatics (OHDSI) is a community developed from the Observational Medical Outcomes Partnership (OMOP) [39-42]. The OMOP CDM is dedicated to observational studies, medical product safety surveillance, comparative effectiveness research, and patient-level predictive modeling. In this context, the OHDSI community shares methods and tools for the use of the OMOP CDM, which standardizes the structure and vocabulary of observational data. Around 2000 collaborators from 74 countries were involved in the OHDSI community in mid-2022 [43]. Analyses could be successfully applied on this model and be used at different data sites around the world [44,45].

Beside clinical data tables, which are appropriate for the storage of individual low-level records (ie, procedure_occurrence, condition_occurrence, and measurement), the OMOP CDM was extended with 5 tables to store derived elements [46]. In particular, the EPISODE table stores the abstracted episodes of care previously defined [47,48] and allows the extraction of chemotherapy episodes from drug records in order to compare anticancer treatment trajectories [49].

Feature extraction methods are poorly described when applied to compute secondary information from retrospective databases. They also lack an approach to store features in a persistent way in a data warehouse. The purpose of this article is to propose a standardized description of the steps and transformations that could help researchers to implement and document feature extraction, and improve the reproducibility of retrospective studies. It also includes identifying how features could be stored in the schema of a data warehouse implemented with the OMOP CDM.

## Methods

### Overview

This study involved the following 3 main steps: (1) the collection of relevant study cases that applied feature extraction and were based on the automatic and secondary use of data; (2) the standardized description of the feature extraction process, including the concepts, their characteristics, and the methods that were common to the study cases; and (3) the proposal of convenient tables to store features in the OMOP CDM.

### Ethics Approval

This study did not require ethics approval as no personal data were collected and no interventions were implemented.

### Collection of Study Cases

We were seeking examples of retrospective observational studies for which feature extraction operations had to be implemented. These studies did not need to be conducted for a specific field of research, during a defined time period, or using a particular data model. The prerequisite was to have transformed raw data into usable information and to be able to describe the process. We focused on studies performed with structured data and did not investigate feature extraction from unstructured data such as text, images, videos, or sound. We contacted researchers from 7 teams involved in data reuse in France between September 1, 2021, and December 31, 2021.

We conducted individual interviews and obtained handwritten notes. The researchers were asked to describe (1) the objective of the study, (2) the database they used (ie, claims or clinical database), (3) the nature of the data and the terminologies, (4) the difficulties they encountered when extracting information from raw data, (5) the features they had to extract to achieve the objectives of the study, (6) the use they made of the features in the study (ie, inclusion criteria, explanatory variables, or response variables), and (7) the steps that composed the feature extraction and the parameters that characterized the features.

The inclusion criteria define the characteristics that subjects must have to be included in a study. They usually include age, type and stage of a disease, and surgical procedure. The response variable is the target of a question in the study or experiment. It is usually survival, length of hospital stay, recovery, or complication of a disease. The explanatory variable is that variable whose changes might affect the response variable. It may be exposure to an event or to a treatment.

The studies were carried out on the following 2 types of databases: claims databases and hospital clinical databases. These 2 sources are relational databases with a tabular format. Each table contains only 1 entity (eg, patients, stays, and diagnoses), and each row corresponds to 1 record. The tables are linked together by the mechanism of foreign keys, allowing the identification of all the data of a patient or a stay, whatever the category. Most of the columns are structured data (ie, 1 type and 1 value per cell). These databases are usually queried using the SQL language. They can then be processed with programming languages, such as R and Python, to recalculate new essential information or to adapt the structure of the data to be able to analyze them more easily.

The claims databases were the French national hospital discharge database, referred to as *Programme de médicalisation des systèmes d'information* (PMSI) [50], and the French national claims database, referred to as *Système National des Données de Santé* (SNDS) [51]. These nationwide databases collect standardized discharge reports for all inpatient stays in French nonprofit or for-profit hospitals. They include individual-level data about the dates of admission and discharge, the hospital code number, the sector code and outcome (ie, discharge, hospital transfer, and death), social demographics (ie, gender, age, and place of residence), diagnoses, and medical procedures performed during the hospital stay. The diagnoses are coded according to the French version of the International Statistical Classification of Diseases and Related Health Problems, 10th Revision (ICD10). The medical procedures are documented according to the *Classification Commune des Actes Médicaux* (CCAM). In addition to these data, the SNDS database includes consumption of care outside the hospital (ie, pharmacy visits, general medical reimbursements, and nursing care). Prescribed medications are documented with the Anatomical Therapeutic Chemical (ATC) system, an international classification system, or with the *Code Identifiant de la Présentation* (CIP13).

The clinical databases were local hospital data warehouses collecting all information about laboratory results, medical procedures, diagnoses, and types of medical units and transfers between them. Two databases included the details of anesthesia procedures (ie, the steps of the surgical procedures, drug administrations, and signals recorded by the equipment in the operating room, eg, mean arterial pressure, heart rate, and tidal volume) [52]. In these databases, vocabularies are local terminologies developed by the software editor and updated by the physician during practice. They cover drugs, measurements, and steps of the surgical procedure. The last database was the Medical Information Mart for Intensive Care III database, a large open-source medical record database of critical care stays, publicly available in PhysioNet [53,54]. Diagnoses are documented with the International Statistical Classification of Diseases and Related Health Problems, 9th Revision (ICD9), and the procedures are documented with the Current Procedural Terminology.

### Standardized Description of the Feature Extraction

In the second step, we performed a hierarchical analysis of the task (HAT) [55]. A HAT allows an understanding of the tasks that users need to accomplish in order to achieve certain goals. These tasks may be decomposed into several levels of subtasks, up to having atomic operations. In this study, we carried out a HAT to (1) understand the steps and transformations that the researchers had to implement to transform raw data into features and (2) identify the successive states of data, from raw data to features, describing the complexity and time dependency.

To do so, we asked them to describe the raw data they had at the beginning, and which were the different transformations they had to chain to obtain features. At each step, we described the complexity and time dependency. We have illustrated the succession of subtasks for each case study, in collaboration with the researcher involved in the study. From the obtained task descriptions and illustrations, we grouped the tasks according to the types of input and output data. Lastly, we propose a description of these different states and transformations, based on what was common to the study cases.

### Evaluation of Feature Storage Possibilities in the OMOP CDM

In the last part, we studied the existing tables of the OMOP CDM that could allow the storage of features without losing information, that is, with adequate fields. In the reverse case, we would propose new tables to conform to the OMOP standard. We would also define the attributes that would have to respect the OMOP standard and keep track of how features were computed to ensure the reproducibility of the studies.

## Results

### Collection of Study Cases

Among the 15 people we contacted, 3 did not answer and 2 reported not performing feature extraction. Based on the semistructured interviews, we collected 8 retrospective and observational studies from teams in 3 French university hospitals (Amiens, Lille, and Rouen) and the French high authority of health. Two of the studies were multisite studies, 4 used claims databases, and 5 used clinical databases.

The features identified represented different types of variables used for conducting retrospective analyses: inclusion criteria, explanatory variables, and response variables. Generic features were (1) occurrences of diagnoses, medical procedures, and age as inclusion criteria; (2) occurrences of medical procedures, occurrences of drug administrations, and transformations of vital signs as explanatory variables; and (3) hospital and intensive care mortality, hospital stay duration, and passage in intensive care as response variables. The study cases and the more complex features reported by the researchers are described in Table 1.

These various study cases were based on complex (ie, heterogeneous, multidimensional, unbalanced, and time-dependent) raw data. The heterogeneity of these raw data comes from the diversity of the variables involved to extract secondary computed features. The first 5 study cases (SC1-5) used measurements and transformed vital signs (arterial pressure and heart rate) or ventilatory signals (partial pressure of oxygen and tidal volume), SC6 and SC7 used drug administrations, and SC7 used laboratory results. In addition to their heterogeneity, the databases are multidimensional, which implies that the tables that compose them have different dimensions (ie, statistical units). Thus, each patient will have a different number of records in the other tables (procedures, diagnoses, measurements, drugs, etc), depending on the length of hospital stay, the care received, and the duration of follow-up. This number of different records from one patient to the other should however be reduced to one line per statistical unit of the study. Next, the modalities of variables are numerous and unbalanced, that is, each terminology has thousands of codes, some of which are widely used, while others are almost never needed. As a result, at the time of feature extraction, these thousands of codes generate as many columns, with, for example, features reporting the code as absent/present or the number, or reporting the number of times it has been documented. At last, raw data are time-dependent variables, that is, variables that are not necessarily constant over the course of the study.

**Table 1:** Description of study cases involving feature extraction for retrospective observational studies.

Study case	Objective of the study	Features needed to achieve the objectives of the study
SC1: Detection of hyperoxemia in mechanically ventilated patients	To evaluate the effect of hyperoxemia on ICU^a^ mortality, during the first 24 h of ICU stay, in mechanically ventilated patients with septic shock according to the SEPSIS-3 criteria [56]	Explanatory variable: Weighted average of PaO_2_^b^ for mechanically ventilated patients with septic shock according to the SEPSIS-3 criteria. The measurements are recorded at irregular intervals. The signal is reconstructed to give one measurement per second.
SC2: Duration of hypotension during heavy surgery	To evaluate the impact of early blood pressure control in heavy surgeries on in-hospital mortality and length of stay	Explanatory variable: Duration of arterial pressure spent with a drop of 10% from the average value, during the procedure.
SC3: Duration of hypotension during cesarean section with spinal anesthesia	To characterize the effect of hypotension during cesarean section with spinal anesthesia on fetal pain	Explanatory variable: Duration of systolic arterial pressure with a drop of 20% from a reference value between induction and birth for a cesarean section with spinal anesthesia. The reference value is the mean value of the systolic arterial pressure between arrival in the operating room and the induction.
SC4: Heart rate and administration of atropine	To assess the evolution of heart rate before and after the administration of atropine (a medication used to treat bradycardia)	Explanatory variables: The median, minimum, and maximum values of heart rate are computed during 2 periods of 10 minutes, designed around the administration of atropine.
SC5: Compliance with ventilatory guidelines	To evaluate whether the recommendations in terms of ventilation in the operating room have been carried out [57]	Explanatory variable: End-tidal volume <8 mL/kg of ideal body weight during surgery.
SC6: Potentially inappropriate medications	To measure the impact of a therapeutic optimization intervention included in an integrated care pathway on PIM^c^ prevalence and on hospital readmission in frail older people	Explanatory variable: Number of drug administrations from the French Laroche list [58] (potentially inappropriate medications) in the 90 days preceding the hospitalization. Number of drug administrations from the French Laroche list in the 90 days following the hospitalization.
SC7: Drug-drug interactions	To estimate the probability of the occurrence of INR^d^ changes for each DDI^e^ rule involving VKA^f^ [59]	Explanatory variable: Administration of VKA with another drug defined in a DDI rule. Raw ATC^g^ codes are mapped to wider categories by taking into account the active substances and the administration route. The period of interest started the day after the 2 drugs had been administered together and ended 4 days after the first of the 2 drugs was discontinued. Response variable: VKA potentiation with at least one value of INR ≥5 or VKA inhibition with at least one value of INR ≤1.5.
SC8: Compliance with guidelines for COPD^h^ patients	To assess the percentage of suspect COPD patients having functional respiratory exploration for diagnosis	Explanatory variable: Suspect COPD patients defined as patients aged more than 40 years with one of several of the following treatments: bronchodilators, 3 antibiotic therapies for respiratory infection, or nicotinic substitutes.

^a^ICU: intensive care unit.

^b^PaO_2_: partial pressure of oxygen.

^c^PIM: potentially inappropriate medication.

^d^INR: international normalized ratio.

^e^DDI: drug-drug interactions.

^f^VKA: vitamin K antagonist.

^g^ATC: Anatomical Therapeutic Chemical.

^h^COPD: chronic obstructive pulmonary disease.

### Standardized Description of the States and Transformations Related to Feature Extraction

**Figure 1:**
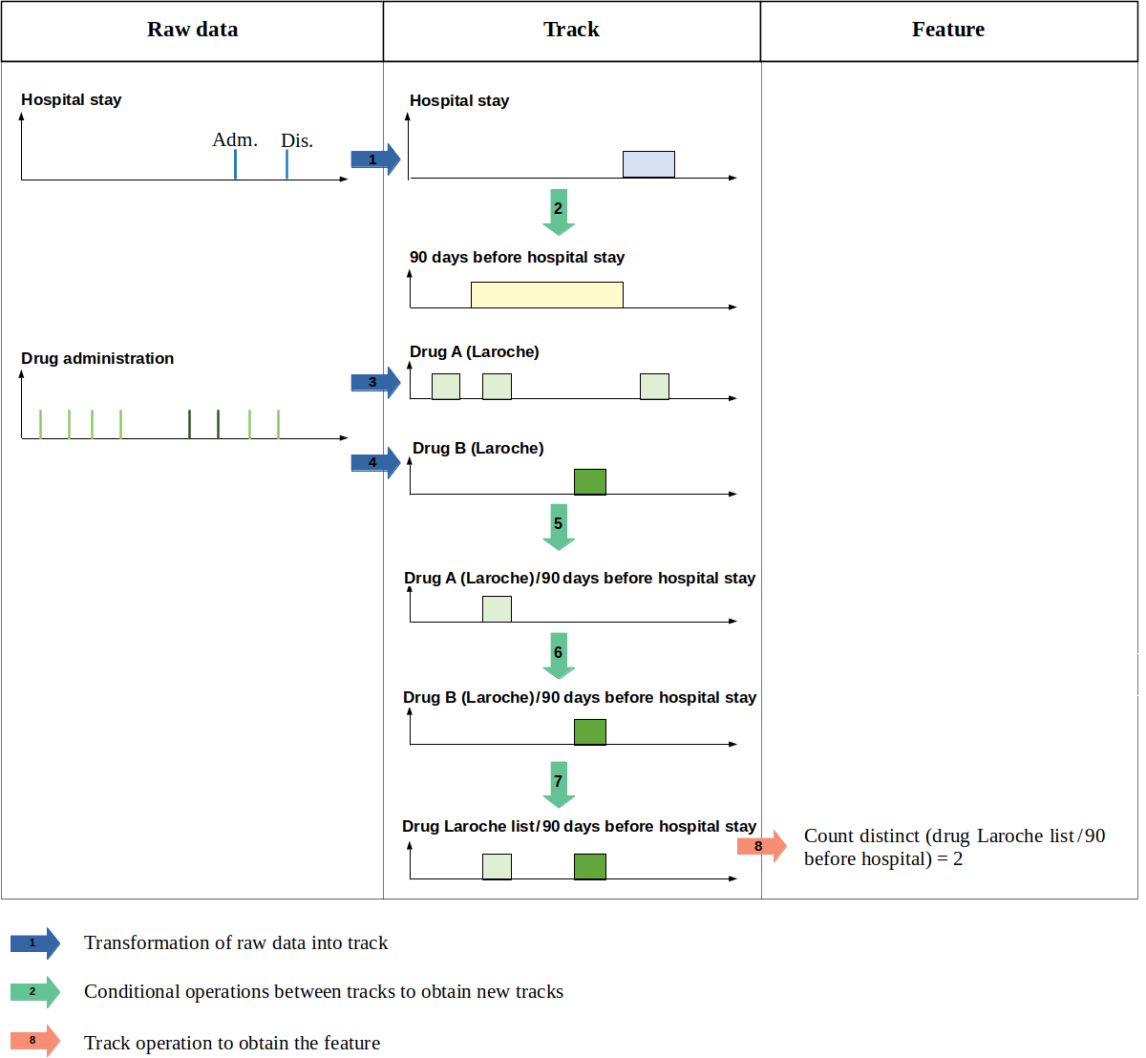
Standardized description of study case 6.

Figure 1 provides the complete description of SC6. First, raw records of administrative data were transformed into a new type of record corresponding to the occurrence of hospital stay (step 1). We will refer to this period as “track” in the rest of the manuscript. Then, this track was transformed to obtain a second track representing the 90 days before hospital stay (90_days) (step 2). Drug administrations included in the Laroche list were identified from raw records, and the periods of administration of drug A and drug B were computed based on the dates of administration and the duration of treatment, in steps 3 and 4, respectively. Similar tracks were computed for all drugs included in the Laroche list, but for the clarity of the figure, we have chosen to illustrate only the first 2 drugs. After these 4 steps, comparisons between tracks were realized successively. This allowed comparisons of the tracks of administration of drug A and drug B to track 90_days, in steps 5 and 6, respectively. The results were joined in a common track to obtain the tracks of the administration of Laroche list items during track 90_days (step 7). Lastly, the number of distinct items was counted to obtain the final feature, that is, the number of drugs from the Laroche list administered in the 90 days preceding the hospital stay.

Table 2 summarizes these transformations, as well as the input and output data of each transformation. Standardized descriptions of all other study cases and feature extraction processes are available in Multimedia Appendix 1 and Multimedia Appendix 2.

**Table 2:** Input data, transformations, and output data for each step involved in the feature extraction of study case 6 (potentially inappropriate medications).

Step	Input data	Transformation	Output data
1	Raw data: Hospital stay	Selection of fields “admission date” and “discharge date”	Track: Hospital stay
2	Track: Hospital stay	Computing the previous 90 days	Track: 90 days before hospital stay
3	Raw data: Drug administration	Selection of drugs included in the Laroche list	Track: Drug A
4	Raw data: Drug administration	Selection of drugs included in the Laroche list	Track: Drug B
5	Track: 90 days before hospital stay + Track: Drug A	Intersection of the 2 tracks	Track: Drug A (Laroche)/90 days before hospital stay
6	Track: 90 days before hospital stay + Track: Drug B	Intersection of the 2 tracks	Track: Drug B (Laroche)/90 days before hospital stay
7	Track: Drug A (Laroche)/90 days before hospital stay + Track: Drug B (Laroche)/90 days before hospital stay	Union of the 2 tracks	Track: Drug Laroche list/90 days before hospital stay
8	Track: Laroche list/90 days before hospital stay	Count distinct (drug Laroche list/90 days before hospital stay)	Feature: Number of drugs from the Laroche list prescribed in the 90 days before hospital stay

### States and Transformations

**Figure 2:**
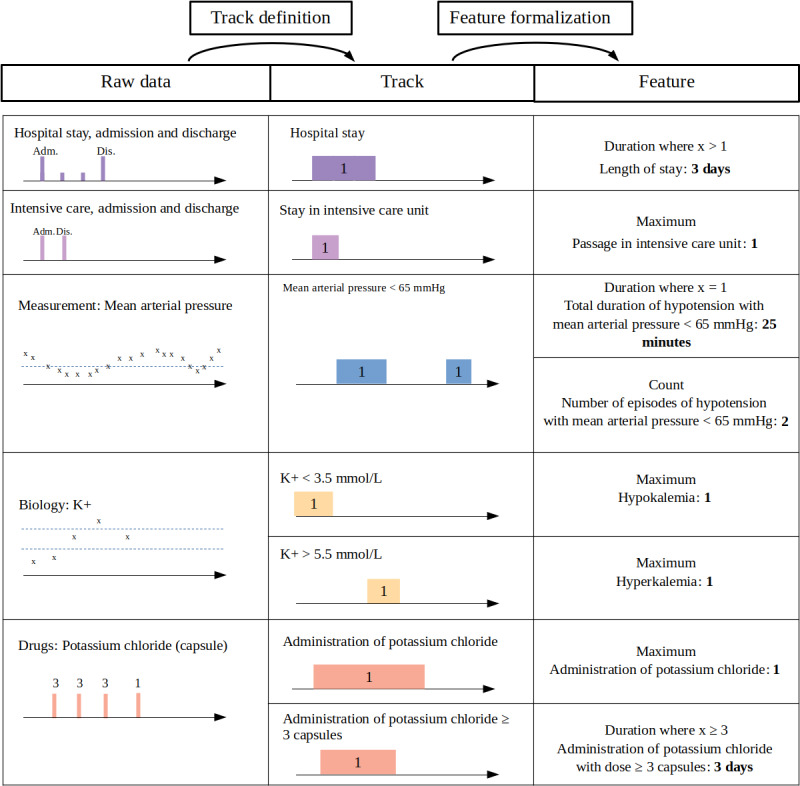
Feature extraction process transforming raw data into features.

Based on the study cases and the HAT, we identified that data went through 2 states (track and feature) and benefited from 2 transformations (track definition and track aggregation). Table 3 summarizes the differences between the raw data, track, and feature, as well as the definitions of the 2 transformations. The whole process of feature extraction is illustrated for several types of raw data in Figure 2, and is fully described below.

The step of *track definition* aims at reducing the dimensions of raw data to the statistical unit of the study, which is the element of the population on which the statistical study is conducted. The statistical unit may refer to not only a patient, but also a hospital, hospital stay (SC6), specialized unit stay (SC1), or a procedure (SC2, SC3, SC4, and SC5), depending on the purpose of the study. During track definition, the data may be rebuilt or computed based on operations such as the selection of variables and values, the mappings between codes of terminologies (SC6 and SC7), the detection of the passage of values beyond a threshold (SC2 and SC3), or the application of any other expert rule (SC5, SC6, and SC7).

*Track* is an intermediate state between raw data and features. It results from the first operation and remains a time-dependent signal, defined by a statistical unit, a type of track, a value, or a set of values. The type of track may be the passage in a care unit, the administration of a drug, a health condition characterized by a diagnosis, or a heart rate signal. The value represents the track state, with a binary value for an on/off state or a quantitative value for a signal. Conditional operations may also be applied between tracks to generate new ones (eg, for detecting the simultaneous administration of 2 drugs). Based on this definition, Table 4 presents the tracks for the 8 study cases.

The step of *track aggregation* extracts final information from tracks during a specified period of interest. The extraction method reduces the multidimensionality and releases from the dependence on time. These methods are usual statistical functions (eg, minimum, maximum, mean, median, count, duration, and delay).

The *period of interest* is defined by a start date and an end date, which may come from different sources as follows: the administration of a drug, the step of a procedure, the visit with a health care professional, or the visit to a health care unit. For each date, there could be more than one candidate event. For example, in SC3, the start of the anesthesia procedure may be documented with 4 different events as follows: induction event, hypnotic administration, intubation, and mechanical ventilation. In the same way, the end of the anesthesia procedure may be defined by the following 2 events: extubation or the end of the anesthesia event. In this case, a priority rule based on expert knowledge or an aggregation operation (first or last event) selects the main event. Lastly, a time interval may be added to the start and end dates of the period to create an artificial period as follows: the 90 days preceding or following hospitalization (SC6).

At the end of the process, *feature* is a single value associated with a label (the feature name). In a feature, time is implicit and is no longer formalized by a date in the record. It may be sometimes represented in the name of the variable, with, for example, the mean value of arterial pressure before induction (eg, mean_map_before_induction). It may also be represented in the value of the feature itself (eg, for a delay or a duration). The feature depends greatly on the context of the study; thus, in SC2 and SC3, the same raw signal produces 2 distinct features that depend on the extraction methods and the periods of interest. Table 5 describes the features identified in our 8 study cases, according to the statistical unit, period, signal, and extraction method.

**Table 3:** Definitions and comparisons of the states and transformations involved in the feature extraction.

States and transformations	Description	Example	Time dimension	Complexity
Raw data (state)	Heterogeneous, multidimensional, and time-dependent low-level clinical data: demographic data, patient flow, laboratory results, drug administrations, procedures, diagnoses, and measurements. The time dimension is always beside the value as an attribute.	Raw measurements of mean arterial pressure	Yes	Yes
Track definition (transformation)	Reduction of the initial dimensions to the statistical unit and standardization of the data representation through an infinite possibility of operations with high expert knowledge. Conservation of the time dimension. Conditional operations may be performed on tracks to generate new tracks.	Resampling of the signal	Yes	Reduced
Track (state)	Homogeneous and time-dependent signal, defined by a homogeneous statistical unit, a type of track, and a set of time-stamped values. The time dimension remains beside each track.	Resampled signal with one measurement per second	Yes	No
Track aggregation (transformation)	Reduction of the time dimension: a period of interest, a track, and an extraction method based on a finished number of operations (minimum, maximum, median, sum, count, etc). The time dimension is reduced to obtain a single value, with time embedded in the variable name or inside the value.	Aggregation (minimum and mean values) of measurements recorded between the start and end of the anesthesia procedure	Reduced	No
Feature (state)	Time-independent high-level information with dimensionality identical to the statistical unit of the study, defined by a label and a value. The time dimension has become implicit in the value (eg, in a delay or a duration) or name of the variable (eg, a value at day 1).	Minimum and mean values of mean arterial pressure during the anesthesia procedure	Implicit	No

**Table 4:** Definition of tracks used in the study cases.

Study case and statistical unit			Track	Value(s)
**SC1: Hyperoxemia in mechanically ventilated patients**				
	ICU^a^ stay	First 24 hours of ICU stay for mechanically ventilated patients with septic shock		ICU stay=1
	ICU stay	Resampled PaO_2_^b^		PaO_2_ repeated measurements
**SC2: Duration of hypotension during general anesthesia**				
	Heavy surgery	General anesthesia procedure		General anesthesia procedure=1
	Heavy surgery	Average value of mean arterial pressure		Average value
	Heavy surgery	Episode of mean arterial pressure below 90% of the average value		Episode=1
**SC3: Duration of hypotension during cesarean section with spinal anesthesia**				
	Cesarean section with spinal anesthesia	Arrival in the operating room to induction of anesthesia		Reference period=1
	Cesarean section with spinal anesthesia	Induction of anesthesia to birth		Spinal anesthesia=1
	Cesarean section with spinal anesthesia	Average value of the systolic arterial pressure between arrival in the operating room and induction of anesthesia		Average value
	Cesarean section with spinal anesthesia	Episode of systolic arterial pressure below 80% of the average value		Episode=1
**SC4: Heart rate and administration of atropine**				
	Administration of atropine	Before administration of atropine		Before=1
	Administration of atropine	After administration of atropine		After=1
**SC5: Compliance with ventilatory guidelines**				
	Anesthesia procedure with mechanical ventilation	Surgery		Surgery=1
**SC6: Potentially inappropriate medications**				
	Hospital stay	Before hospital stay		Before hospital stay=1
	Hospital stay	After hospital stay		After hospital stay=1
	Hospital stay	Administration of drug X from the Laroche list		Drug X=1
**SC7: Drug-drug interactions**				
	Patient	Administration of drug X (raw code)		Drug X=1
	Patient	Administration of a drug family (ATC^c^ category)		ATC category=1
	Patient	Concomitant administration of a VKA^d^ with a drug defined in a DDI^e^ rule		Concomitant administration=1
	Patient	INR^f^ ≥5		Episode of INR ≥5
	Patient	INR ≤1.5		Episode of INR ≤1.5
	Patient	Concomitant administration of a VKA with a drug defined in a DDI rule and INR ≥5		VKA potentiation=1
	Patient	Concomitant administration of a VKA with a drug defined in a DDI rule and INR ≤1.5		VKA inhibition=1
**SC8: Compliance with guidelines for COPD patients**				
	Patient	Administration of one of several drugs among bronchodilators or nicotinic substitutes (ATC codes)		Drug X ≥1
	Patient	Administration of 3 antibiotic therapies for respiratory infection (ATC codes)		Drug X ≥3
	Patient	Exposure to at least one of the drugs specific to suspected COPD^g^		Exposure to COPD-specific drugs=1
	Patient	Induction of spirometry or functional respiratory exploration		Episode=1

^a^ICU: intensive care unit.

^b^PaO_2_: partial pressure of oxygen.

^c^ATC: Anatomical Therapeutic Chemical.

^d^VKA: vitamin K antagonist.

^e^DDI: drug-drug interaction.

^f^INR: international normalized ratio.

^g^COPD: chronic obstructive pulmonary disease.

**Table 5:** Definitions of the characteristics for each feature of the study cases.

Study case	Statistical unit	Period	Track	Extraction method
SC1: Hyperoxemia in mechanically ventilated patients	ICU^a^ stay	First 24 hours of ICU stay for mechanically ventilated patients with septic shock	Resampled PaO_2_^b^	Weighted average
SC2: Hypotension during anesthesia	General anesthesia procedure	Anesthesia period	Mean arterial pressure	Sum of the duration of episodes of mean arterial pressure with a drop of 10% from the reference value
SC3: Duration of hypotension during cesarean section with spinal anesthesia	Cesarean section with spinal anesthesia	Anesthesia period	Systolic arterial pressure	Total duration of systolic arterial pressure below 80% of the reference value
SC4 :Heart rate and administration of atropine	Administration of atropine	Periods of 10 minutes before and after the administration of atropine	Heart rate	Median, minimum, and maximum values of heart rate
SC5: Compliance with ventilatory guidelines	Anesthesia procedure with mechanical ventilation	Surgery period	End-tidal volume	Mean end-tidal/ideal body weight >8
SC6: Potentially inappropriate medications	Hospital visit	Before hospital stay; after hospital stay	Administration of medications	Count of inappropriate drug administration according to the French Laroche list.
SC7: Drug-drug interactions	Patient	Day after the 2 drugs have been administered together and until 4 days after the first of the 2 drugs was discontinued.	Concomitant administration of a VKA^c^ with a drug defined in a DDI^d^ rule and INR^e^ ≥5. Concomitant administration of a VKA with a drug defined in a DDI rule and INR ≤1.5.	Count of VKA potentiation. Count of VKA inhibition.
SC8: Compliance with guidelines for COPD^f^ patients	Patient	Year following exposure to one of the drugs specific to COPD	Administration of medications	Count of the administration of drugs specific to COPD Binary indicator of FRE^g^ induction

^a^ICU: intensive care unit.

^b^PaO_2_: partial pressure of oxygen.

^c^VKA: vitamin K antagonist.

^d^DDI: drug-drug interaction.

^e^INR: international normalized ratio.

^f^COPD: chronic obstructive pulmonary disease.

^g^FRE: functional respiratory exploration.

### Evaluation of Feature Storage Possibilities in the OMOP CDM

Five tables already exist in the OMOP CDM (DRUG_ERA, DOSE_ERA, CONDITION_ERA, EPISODE, and EPISODE_EVENT) for storing elements derived from raw data [46]. These tables cover the storage of spans of time when the patient is exposed to a specific drug ingredient (DRUG_ERA), when the patient is exposed to a constant dose of a specific drug ingredient (DOSE_ERA), or when the patient is assumed to have a given condition (CONDITION_ERA). These existing tables are suitable for pharmacoepidemiology studies with the comparison of periods of drug exposure and the resulting adverse events or evolution of the disease. The studies require only diagnosis and medication data from the tables CONDITION_OCCURRENCE and DRUG_EXPOSURE [39].

However, other types of data also need to be retransformed to obtain usable information for statistical analysis (in particular, procedures, measurements, biology results, or any types of steps in patient care). At this point, 2 alternatives allow other types of derived elements to be stored. The first approach involves adding an era table for each raw information that can be transformed into an era (ie, a measurement era, procedure era, biology era, etc). The second approach involves proposing a generic era table that would cover all types of raw data. With these 2 approaches, there would still be a lack of storage for the final features, which do not have the same structure as eras or episodes, since they are only an association of a value and a label, independent of time.

For this reason, on the one hand, the table TRACK could complement the model and store intermediate data (ie, all types of tracks and eras), which would ultimately be used to compute features, and on the other hand, the table FEATURE could extend the OMOP CDM for storing secondary computed data from measurements, procedures, observations, and stays, which would be used for the analysis and would need to be stored on a long-term basis.

**Figure 3:**
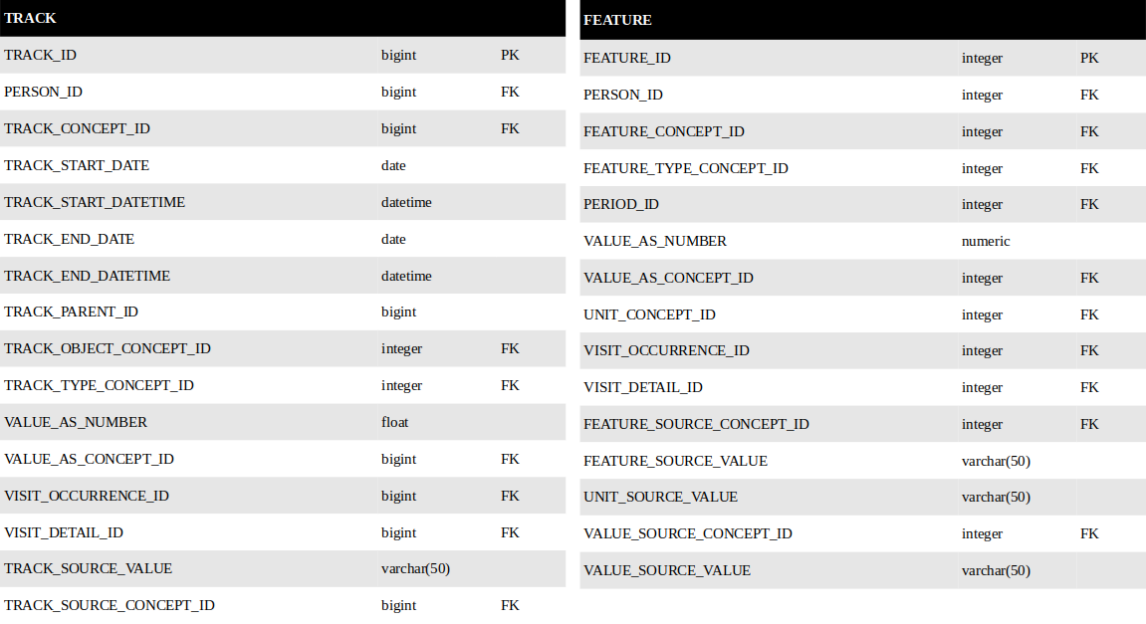
Data model for the storage of periods and features in a relational database, compliant with the Observational Medical Outcomes Partnership (OMOP) common data model. FK: foreign key; PK: primary key.

These 2 new conceptual tables are illustrated in Figure 3. They comply with the OMOP guidelines in terms of field name and table organization [60]. For both tables, foreign keys reference the person, the visit, the visit details, the main concept (TRACK_CONCEPT_ID and FEATURE_CONCEPT_ID), and the type of this concept (TRACK_TYPE_CONCEPT_ID and FEATURE_TYPE_CONCEPT_ID). Similarly, the 2 tables provide core fields to store continuous values (VALUE_AS_NUMBER) or categorical values (VALUE_AS_CONCEPT_ID). The specificity of TRACK involves the preservation of the time dimension through the fields TRACK_START_DATE and TRACK_END_DATE. In the FEATURE table, in the case where a patient could present the same feature several times (eg, on different days), a foreign key to the EPISODE table allows differentiation of the occurrences of a feature [47]. Both tables also have the usual fields to store the source values expressed with local vocabularies.

## Discussion

### Principal Findings

In this article, we propose a standardized description of the feature extraction process, which is implemented when transforming heterogeneous, multidimensional, and time-dependent raw data into valuable information for conducting observational retrospective studies. The process combines 2 steps (track definition and track aggregation). Track definition aims at transforming raw data into multiple tracks representing the periods of interest or reconstructing a signal. Track aggregation computes usable information from a final track for applying an extraction method during a period of interest. The resulting features are the 1-dimensional and time-independent variables that will be included in the statistical analysis.

By dividing the feature extraction into these 2 steps, the difficulty is managed during track definition. The first step aims at creating tracks, with a common unit adequate for the statistical unit of the study and a homogeneous temporal scale. Tracks then allow the application of an infinite number of complex transformations, such as the mapping of concepts for the detection of drug-drug interactions (SC7). These transformations require great expertise with regard to the data and are mainly implemented on a custom basis. On the contrary, track aggregation is a very simple operation, with a finite number of possibilities.

### Strengths of the Study

The definitions of the transformations are based on various cases, and they were carried out on different databases from several centers. Feature extraction is the algorithmic translation of expert knowledge. Our work shows that this process requires the sequencing of several transformations, including, for track definition, the choice of (1) a time-dependent signal or an already available track, (2) a statistical unit, (3) a type of track, and (4) a value or a set of values, and track aggregation is the final transformation based on (5) a track, which is performed during (6) a period of interest and involves (7) an extraction method. The formalization and documentation of these 7 items should enhance the reproducibility of studies and the sharing of features between collaborators, by removing the ambiguity about what is being calculated.

### Limits

In this study, we focused on feature extraction based on expert rules and did not take into account feature extraction based on deep learning techniques [61,62]. In this case, although the aim is also to reduce the dimensionality of the source data, there is no need to interpret features, which are often abstract and designed to result in the best prediction model without being interpreted [62]. Recent advances in natural language processing [63-65] could be leveraged to automatize the extraction of relevant clinical features from clinical text [66]. Once the feature of interest has been well defined, a small annotation campaign should be conducted to fine-tune and evaluate pretrained model performances. Afterwards, the extracted feature can be integrated in our workflow as a new structured piece of information. The impressive results of large language models suggest that a few labeled examples are sufficient to fine-tune these models [67]. Three limitations must be explored before using these models. First, due to the variability of the wordings of clinical concepts, it has not been proved that a large language model can capture every targeted feature. Second, the computing intensiveness is incompatible with large-scale information retrieval. Third, the ability to conduct quick targeted annotation campaigns for precise clinical terms requires appropriate tooling and processes. We have not provided any use cases involving text. However, both tracks and features could be constructed from, for example, the presence of a symptom or the reporting of a scale in a consultation report. Such extraction from raw text raises the question of the automatic detection of specific concepts in text and the performance of the tools used for this.

Although some features, such as the length of stay, are generic and frequently used, the majority remain dependent on the study context. The period of interest and the extraction method are proxies for what is expected by the clinician or researcher, and the feature would need to be manually evaluated to ensure its validity [49].

Even if SNOMED CT (Systematized Nomenclature of Medicine - Clinical Terms) and ICD10 propose aggregate concepts, such as “Hypotension following procedure” (SNOMED CT code 16055431000119108), “Decreased mean arterial pressure” (SNOMED CT code 31013001), or “Hypotension” (ICD10 code I95), these concepts are only a part of the label of a feature, and they do not document how to compute the feature or mention the period (ie, surgery, anesthesia, intensive care unit stay, or first day of hospitalization). Standardized concepts that fully document features are yet to be defined in these terminologies.

At present, we cannot judge the generalization of our proposal. However, this study is the first to propose a standardized description of feature extraction from structured databases. The approach remains to be evaluated by comparing it with other study cases, particularly from other countries.

The next step of this project is the implementation of an R package with functions dedicated to the definition and aggregation of tracks. This package would rely on the OMOP CDM and allow reproducibility of feature extraction. Attention will need to be paid to the physical implementation of the 2 tables and, in particular, to the storage of tracks, which can be voluminous and can impact performance with regard to queries and response times. Finally, it would be relevant to implement a data mart with features arranged in columns (when they are still stored in rows in the feature table) to gain time when building tables to construct cohorts.

### Conclusions

We have clarified the process of feature extraction implemented when conducting retrospective observational studies. We identified 2 transformations (track definition and track aggregation) to transform complex raw data into tracks and features. Track definition requires high expertise, but reduces the complexity of data and simplifies the reduction of time dimensionality during track aggregation.

## Appendix

Multimedia Appendix 1 Description of the study cases.

Multimedia Appendix 2 Standardized description of the tracks and features implemented for each study case.

## Abbreviations

ATC: Anatomical Therapeutic Chemical
CDM: common data model
HAT: hierarchical analysis of the task
ICD10: International Statistical Classification of Diseases and Related Health Problems, 10th Revision
OHDSI: Observational Health Data Sciences and Informatics
OMOP: Observational Medical Outcomes Partnership
SNDS: Système National des Données de Santé (French national claims database)
SNOMED CT: Systematized Nomenclature of Medicine - Clinical Terms
