# Supplementary material for: Standardized Description of the Feature Extraction Process to Transform Raw Data Into Meaningful Information for Enhancing Data Reuse: Consensus Study
Source: JMIR Med Inform. 2022 Oct 17;10(10):e38936. doi: 10.2196/38936 (PMC9623460; doi:10.2196/38936)
Supplement: Multimedia Appendix 1 [file medinform_v10i10e38936_app1.docx]

**Multimedia Appendix 1.** Description of the study cases.

| **Study case** | **Source (claims or clinical database)** | **Terminologies** | **Periode** | **Volume** | **Multisite** |
| --- | --- | --- | --- | --- | --- |
| **SC1: Detection of hyperoxemia in mechanically ventilated patients** | Clinical database | Diagnoses : ICD9  Procedures: CPT | 2001-2012 | 488 patients | No |
| **SC2: Duration of hypotension during heavy surgery** | Clinical database for the hypotension and claim database for in-hospital mortaliy and length of stay. | Procedures : CCAM pour les actes inclus Measurement : local terminology | 2019-2021 | 206 patients | No |
| **SC3: Duration of hypotension during cesarean section with spinal anesthesia** | Clinical database | Local terminology | 2011-2016 | 1053 patients | No |
| **SC4: Heart rate and administration of atropine** | Clinical database | Local terminology | 2010-2014 | 4626 patients | No |
| **SC5: Compliance with ventilatory guidelines** | Clinical database | Local terminology | 2010-2015 | 166 571 | No |
| **SC6: Potentially inappropriate medications** | Claims database | Diagnoses: ICD10  Drugs: ATC | 2015-2018 | 582 patients | Yes |
| **SC7: Drug-drug interactions** | Clinical database | Diagnoses: ICD10  Drugs: ATC  Biology: IUPAC | 2007-2014 | 156,893 patients | Yes |
| **SC8: Compliance with guidelines for COPD patients** | Claims database | Procedures :CCAM  Diagnoses: CIM10  Drugs: CIP13. | 2011-2017 | 1 485 278 patients with COPD | No |

ATC: Anatomical Therapeutic Chemical. CCAM: Classification Commune des Actes Médicaux (a French terminology for the medical procedures). CIP13 : Code Identifiant de la Présentation, a French terminology for drugs. CPT: Current Procedural Terminology. ICD9: International Statistical Classification of Diseases and Related Health Problems, 9th Revision. ICD10: International Statistical Classification of Diseases and Related Health Problems, 10th Revision. IUPAC: International Union of Pure and Applied Chemistry.
