# Supplementary material for: Standardized Description of the Feature Extraction Process to Transform Raw Data Into Meaningful Information for Enhancing Data Reuse: Consensus Study
Source: JMIR Med Inform. 2022 Oct 17;10(10):e38936. doi: 10.2196/38936 (PMC9623460; doi:10.2196/38936)
Supplement: Multimedia Appendix 2 [file medinform_v10i10e38936_app2.pdf]

SC1: Detection of hyperoxemia in mechanically ventilated patients

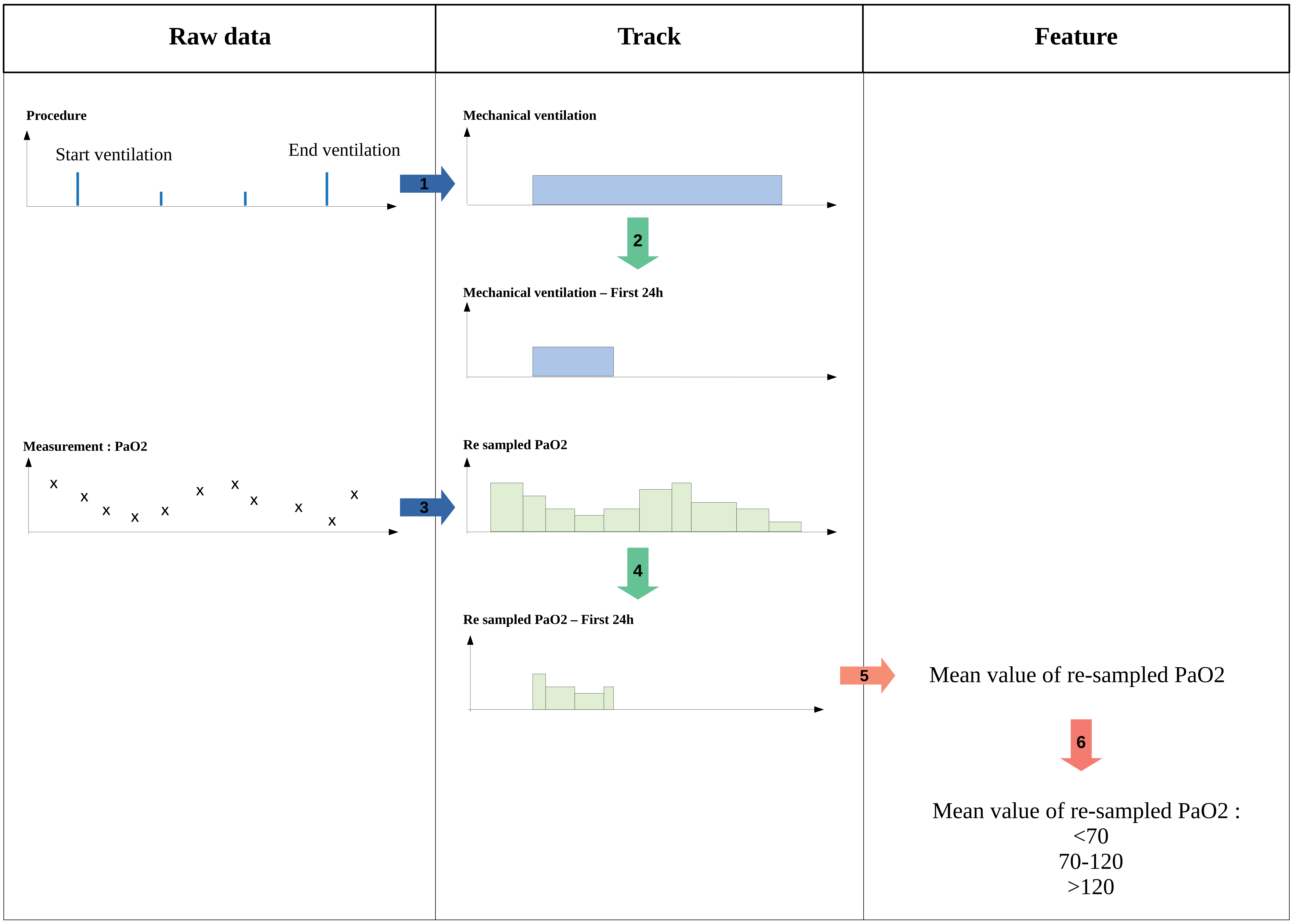

PaO2 : Partial pressure of oxygen

1 Transformation of raw data into track

2 Conditional operations bewteen tracks to obtain new tracks

8 Track operation to obtain the feature

SC1: Detection of hyperoxemia in mechanically ventilated patients

ID	In	Operation	Out
1	Raw table – Procedure	Selection of Fields «Start ventilation data » and «End ventilation »	Track – Mechanial ventilation
2	Track – Hospital stay	Filtering on the first 24 hours	Track – Mechanical ventilation – first 24 hours
3	Raw table – Measurement Pa02	Re-sampling of the signal with 1 measurement / minute	Track – Re-sampled PaO2
4	Track – Mechanical ventilation – first 24 hours<br>+<br>Track – Re-sampled PaO2	Filtering PaO2 in the first 24 hours	Track – Re-Sampled PaO2 in the first 24 hours
5	Track – Re-Sampled PaO2 in the first 24 hours	Computing mean value	Feature – Mean value of PaO2 in the first 24 hours
6	Feature – Mean value of PaO2 in the first 24 hours	Applying threshold	Feature – Classifying as hypoxemia, normoxemia, hyperemia

SC2: Duration of hypotension during general anesthesia

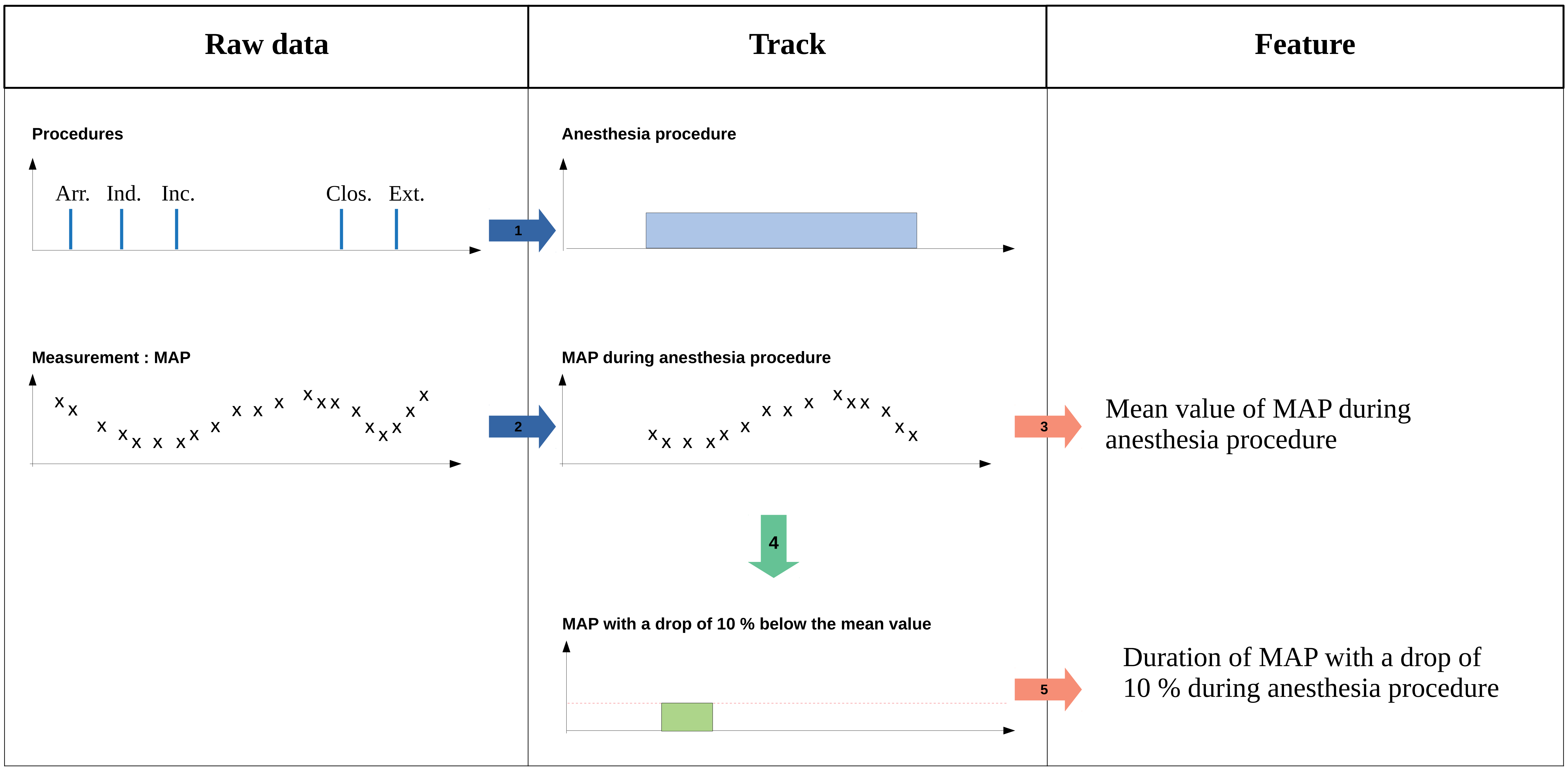

MAP : Mean Arterial Pressure

1 Transformation of raw data into track

2 Conditional operations bewteen tracks to obtain new tracks

8 Track operation to obtain the feature

SC2: Duration of hypotension during general anesthesia

ID	In	Operation	Out
1	Raw table – Procedure	Selection of Fields «Induction» and «Extubation»	Track – Anesthesia procedure
2	Track – Measurement	Filtering the measurements of mean arterial pressure during the anesthesia procedure	Track – Measurements of mean arterial pressure during the anesthesia procedure
3	Track – Measurements of mean arterial pressure during the anesthesia procedure	Computing the mean value	Feature – Mean value of mean arterial pressure during anesthesia procedure
4	Track – Measurements of mean arterial pressure during the anesthesia procedure	Filtering Threshold – Drop below 10 % of mean value	Track – Mean arterial pressure with a drop of 10 % below the mean value
5	Track – Mean arterial pressure with a drop of 10 % below the mean value	Computing duration below a drop of 10 % below the mean value	Feature – Duration of mean arterial pressure with a drop of 10 % during anesthesia procedure

SC3: Duration of hypotension during cesarean section with spinal anesthesia

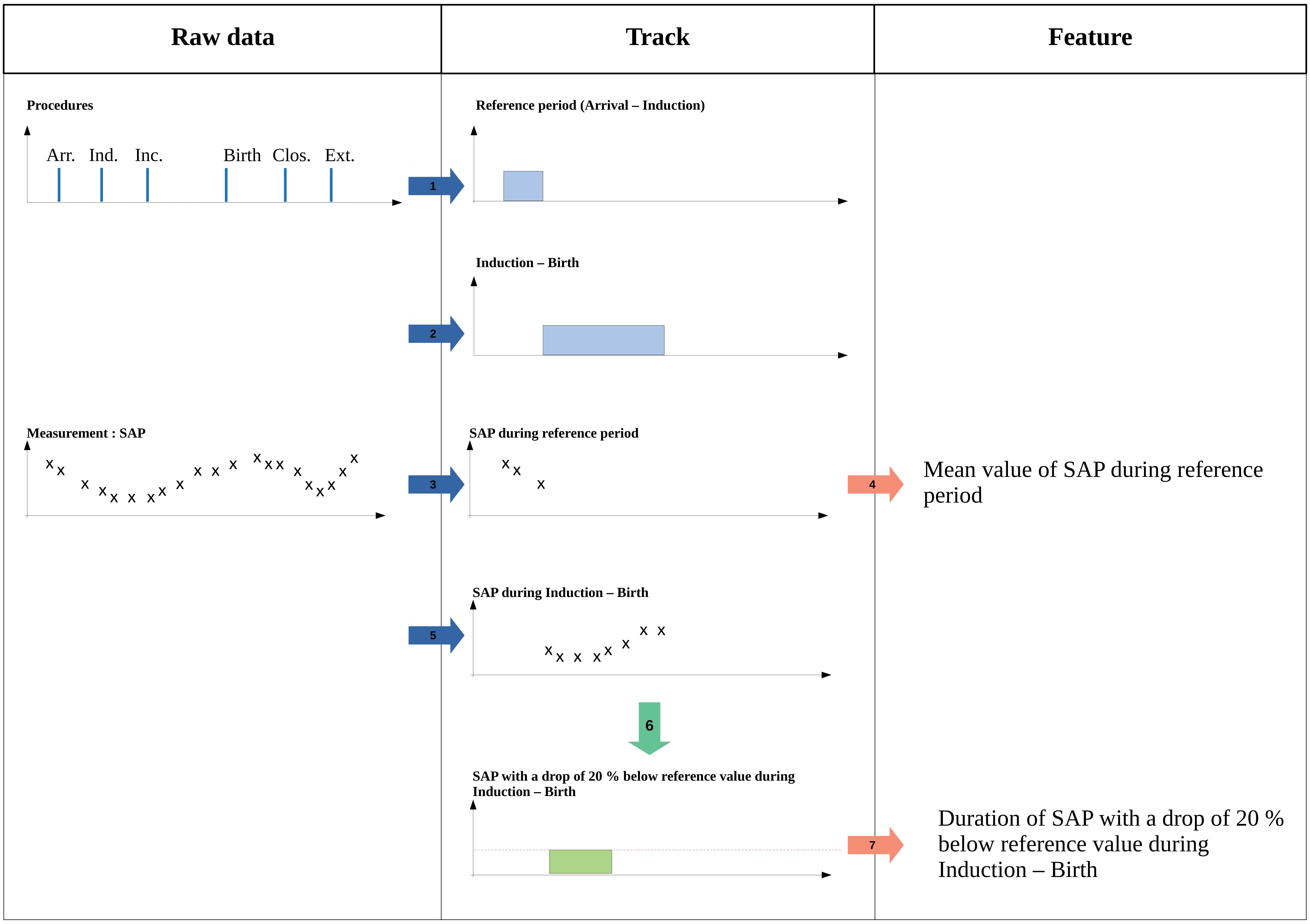

SAP. : Systolic arterial pressure

1 Transformation of raw data into track

2 Conditional operations bewteen tracks to obtain new tracks

8 Track operation to obtain the feature

SC3: Duration of hypotension during cesarean section with spinal anesthesia

ID	In	Operation	Out
1	Raw table – Procedure	Selection of fields «Arrival» and «Induction»	Track – Reference period (Arrival – Induction)
2	Raw table – Procedure	Selection of fields «Induction» and «Birth»	Track – Induction – Birth
3	Raw table – Measurement	Filtering the measurements of systolic arterial pressure during the reference period	Track – Measurements of systolic arterial pressure during the anesthesia procedure
4	Track – Measurements of systolic arterial pressure during the anesthesia procedure	Computing the mean value	Feature – Mean value of systolic arterial pressure during the reference period
5	Raw table – Measurement	Filtering the measurements of systolic arterial pressure during the period Induction – Birth	Track – Measurements of systolic arterial pressure during the period Induction – Birth
6	Track – Measurements of systolic arterial pressure during the period Induction – Birth	Detecting a drop below 20 % of the mean value of systolic arterial pressure during the reference period	Track – Episode of hypotension
7	Track – Episode of hypotension	Computing the duration	Feature – Duration of hypotension

SC4: Heart rate and administration of atropine

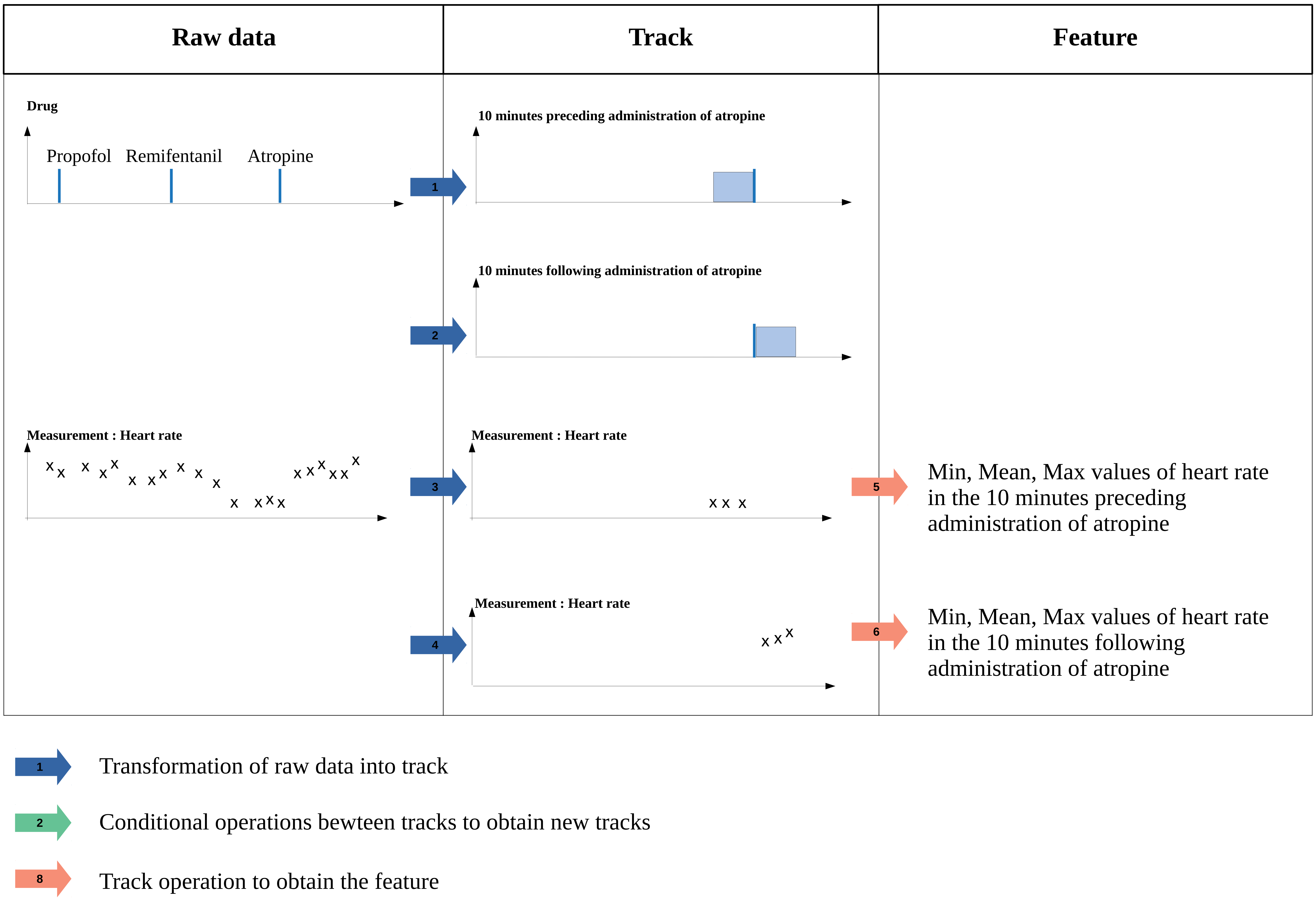

SC4: Heart rate and administration of atropine

ID	In	Operation	Out
1	Raw table – Drug	Selection of administration of atropine	Track – 10 minutes before atropine
2	Raw table – Drug	Selection of administration of atropine	Track – 10 minutes after atropine
3	Raw table – Measurement	Filtering the measurements of heart rate during track – 10 minutes before atropine	Track – Measurements of heart rate during track – 10 minutes before atropine
4	Raw table – Measurement	Filtering the measurements of heart rate during track – 10 minutes after atropine	Track – Measurements of heart rate during track – 10 minutes after atropine
5	Track – Measurements of heart rate during track – 10 minutes before atropine	Computing the min, max, mean values of heart rate during track – 10 minutes before atropine	Feature – Min, Max, Mean value of hear rate during track – 10 minutes before atropine
6	Track – Measurements of heart rate during track – 10 minutes after atropine	Computing the min, max, mean values of heart rate during track – 10 minutes after atropine	Feature – Min, Max, Mean value of hear rate during track – 10 minutes after atropine

SC5: Compliance with ventilatory guidelines

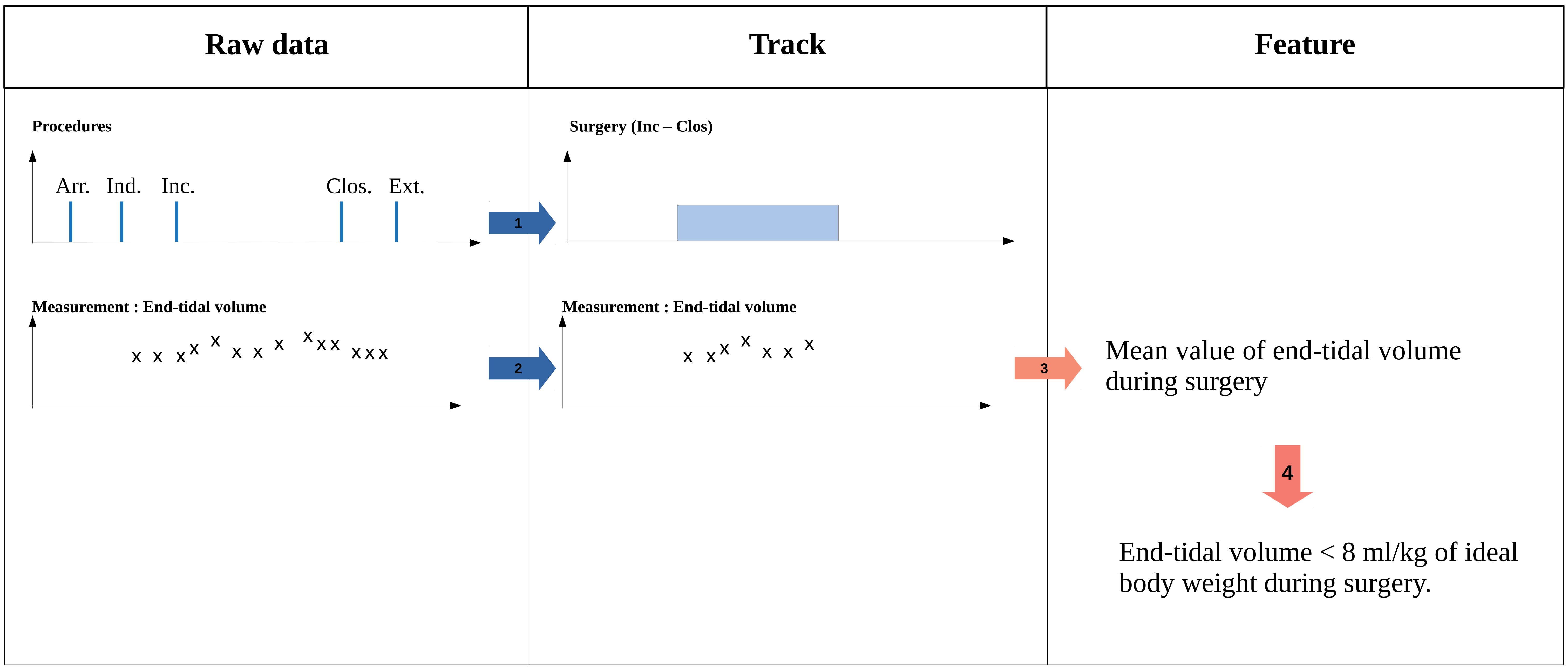

1 Transformation of raw data into track

2 Conditional operations bewteen tracks to obtain new tracks

8 Track operation to obtain the feature

SC5: Compliance with ventilatory guidelines

ID	In	Operation	Out
1	Raw table – Procedure	Selection of Fields «Incision» and «Closure»	Track – Surgery
2	Raw table – Measurement	Filtering the measurement of end-tidal volume during track – surgery	Track – Measurements of end-tidal volume during track – surgery
3	Track – Measurements of end-tidal volume during track – surgery	Computing the mean value of end-tidal volume during track – surgery	Feature – Mean value of end-tidal volume during surgery
4	Feature – Mean value of end-tidal volume during surgery	Applying a threshold at 8 ml/kg of ideal body weight	Feature – Mean value of end-tidal volume < 8 ml/kg of ideal body weight

SC6: Potentially inappropriate medications

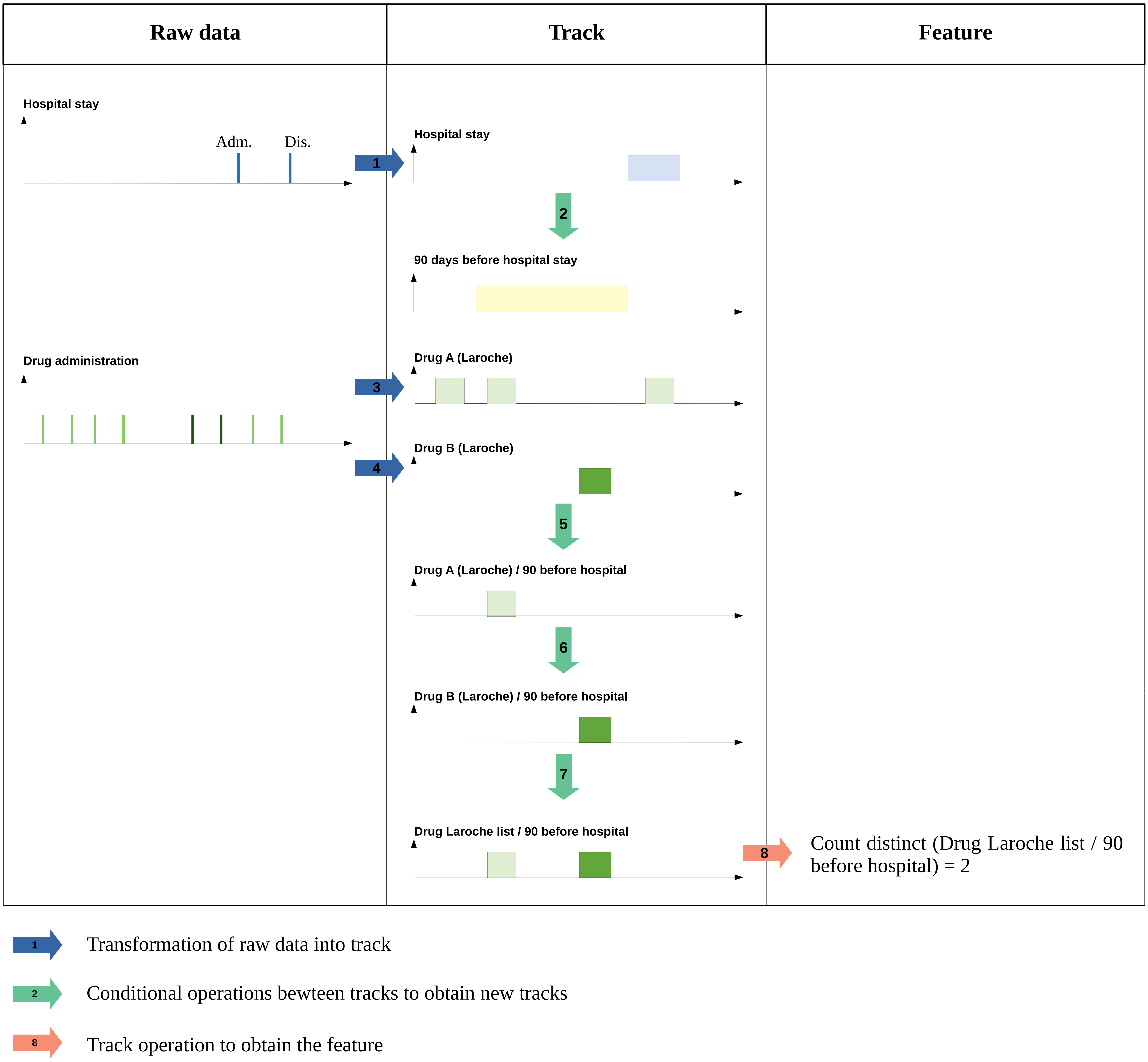

SC6: Potentially inappropriate medications

ID	In	Operation	Out
1	Raw data: Hospital stay	Selection of fields « Admission data » and «Discharge date »	Track : Hospital stay
2	Track : Hospital stay	Computing of previous 90 days	Track 90 days before hospital stay
3	Raw data: Drug administration	Selection of drugs included in the Laroche list	Track Drug A
4	Raw data: Drug administration	Selection of drugs included in the Laroche list	Track Drug B
5	Track : 90 days before hospital stay + Track Drug A	Intersection of the two tracks	Track Drug A (Laroche) / 90 before hospital
6	Track 90 days before hospital stay + Track Drug B	Intersection of the two tracks	Track Drug B (Laroche) / 90 before hospital
7	Drug A (Laroche) / 90 before hospital + Drug B (Laroche) / 90 before hospital stay	Union of the two tracks	Track Drug Laroche list / 90 before hospital
8	Drug Laroche list / 90 before hospital stay	Count distinct (Drug Laroche list / 90 before hospital)	Feature Number of drug from Laroche list prescribed in the 90 days before hospital stay

INR: international normalized ratio
VKA: vitamin K antagonists

SC7: Drug-drug interactions

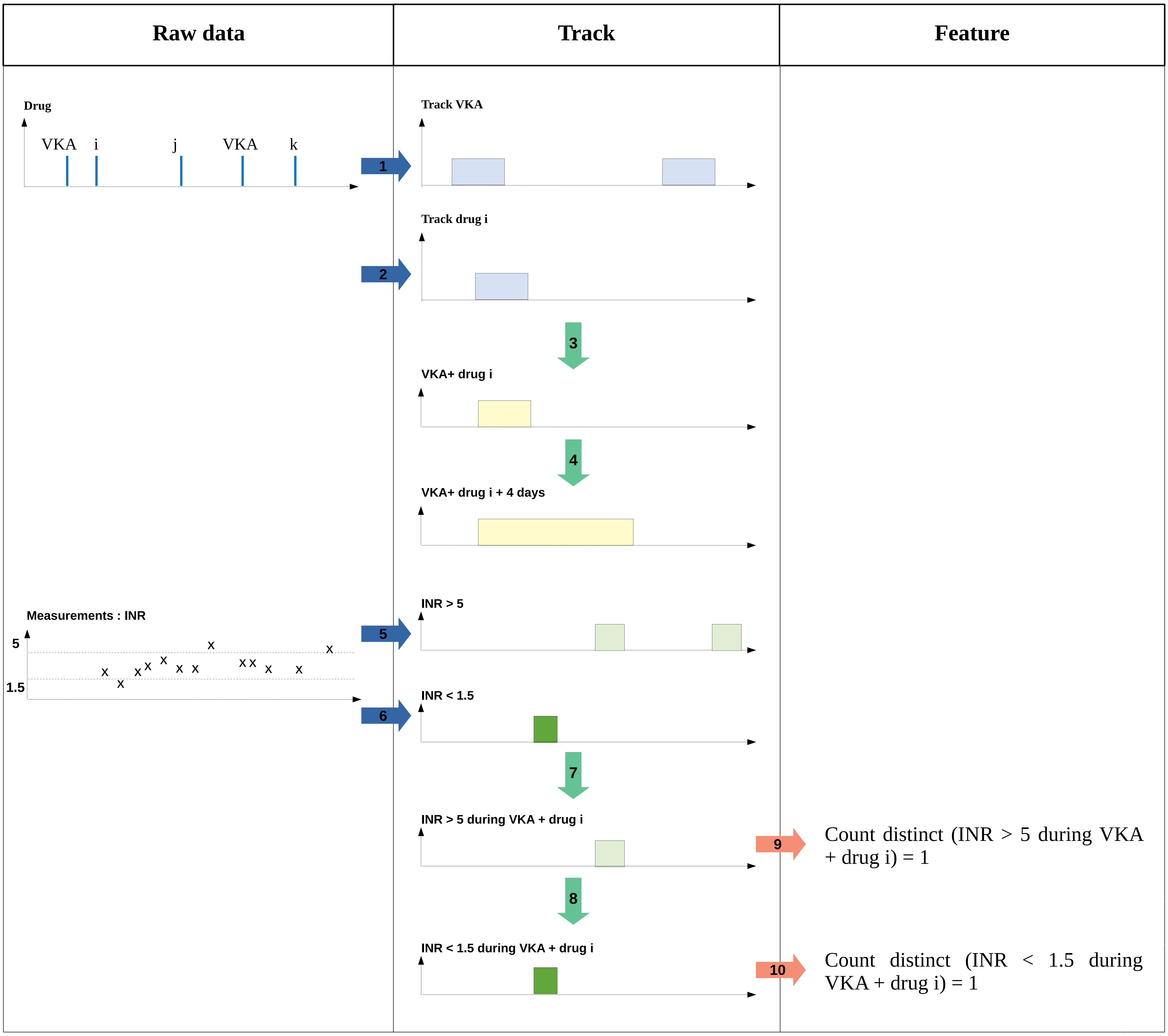

DDI : drug-drug interaction
INR: international normalized ratio
VKA: vitamin K antagonists

1 — Transformation of raw data into track

2 — Conditional operations bewteen tracks to obtain new tracks

8 — Track operation to obtain the feature

SC7: Potentially inappropriate medications

ID	In	Operation	Out
1	Raw data: Drug	Selection of administrations of VKA	Track : Administration of drug VKA
2	Raw data : Drug	Selection of administrations of drug i (defined in a DDI)	Track : Administration of drug i
3	Track : Administration of VKA<br>+<br>Track : Administration of drug i	Intersection of the two tracks	Track : Drug VKA + Drug i
4	Track : Drug VKA + Drug i	Adding of a 4 day delay	Track : Drug VKA + Drug i + 4 days
5	Raw data : Measurement	Detection of INR > 5	Track : INR > 5
6	Raw data : Measurement	Detection of INR < 1.5	Track : INR < 1.5
7	Track : INR > 5<br>+<br>Track : Drug VKA + Drug i + 4 days	Intersection of the two tracks	Track : INR > 5 during period of VKA + drug i
8	Track : INR < 1.5<br>+<br>Track : Drug VKA + Drug i + 4 days	Intersection of the two tracks	Track : INR < 1.5 during period of VKA + drug i
9	Track : INR > 5 during period of VKA + drug i	Count the distinct number of INR > 5 during period of VKA + drug i	Feature : distinct number of INR > 5 during period of VKA + drug i
10	Track : INR < 1.5 during period of VKA + drug i	Count the distinct number of INR < 1.5 during period of VKA + drug i	Feature : distinct number of INR > 5 during period of VKA + drug i

SC8: Compliance with guidelines for COPD patients

Raw data	Track	Feature
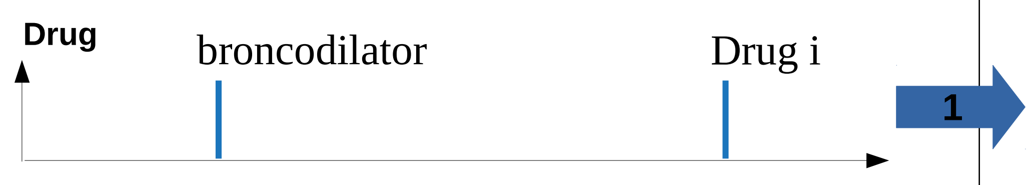	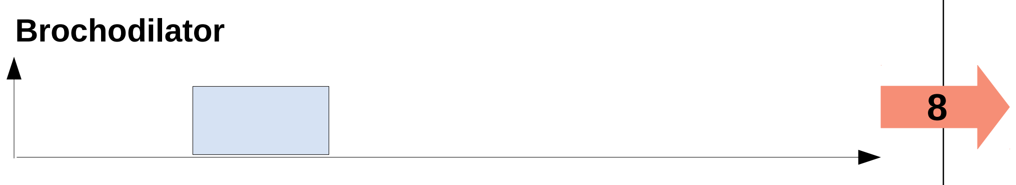	Presence (Bronchodilatator) = 2

1 Transformation of raw data into track

2 Conditional operations bewteen tracks to obtain new tracks

8 Track operation to obtain the feature

SC8: Compliance with guidelines for COPD patients

ID	In	Operation	Out
1	Raw data: Drug	Selection of drug « Bronchodilatator »	Track : Administration of bronchodilatator
2	Track : Administration of bronchodilatator	Presence of bronchodilatator	Feature : Presence of bronchodilatator
